# An Efficient Computational Model for Large-Scale Prediction of Protein–Protein Interactions Based on Accurate and Scalable Graph Embedding

**DOI:** 10.3389/fgene.2021.635451

**Published:** 2021-02-26

**Authors:** Xiao-Rui Su, Zhu-Hong You, Lun Hu, Yu-An Huang, Yi Wang, Hai-Cheng Yi

**Affiliations:** ^1^Xinjiang Technical Institute of Physics and Chemistry, Chinese Academy of Sciences, Ürümqi, China; ^2^University of Chinese Academy of Sciences, Beijing, China; ^3^Xinjiang Laboratory of Minority Speech and Language Information Processing, Ürümqi, China

**Keywords:** large-scale, protein-protein interaction, GraphZoom, weighted graph, graph embedding

## Abstract

Protein–protein interaction (PPI) is the basis of the whole molecular mechanisms of living cells. Although traditional experiments are able to detect PPIs accurately, they often encounter high cost and require more time. As a result, computational methods have been used to predict PPIs to avoid these problems. Graph structure, as the important and pervasive data carriers, is considered as the most suitable structure to present biomedical entities and relationships. Although graph embedding is the most popular approach for graph representation learning, it usually suffers from high computational and space cost, especially in large-scale graphs. Therefore, developing a framework, which can accelerate graph embedding and improve the accuracy of embedding results, is important to large-scale PPIs prediction. In this paper, we propose a multi-level model LPPI to improve both the quality and speed of large-scale PPIs prediction. Firstly, protein basic information is collected as its attribute, including positional gene sets, motif gene sets, and immunological signatures. Secondly, we construct a weighted graph by using protein attributes to calculate node similarity. Then GraphZoom is used to accelerate the embedding process by reducing the size of the weighted graph. Next, graph embedding methods are used to learn graph topology features from the reconstructed graph. Finally, the linear Logistic Regression (LR) model is used to predict the probability of interactions of two proteins. LPPI achieved a high accuracy of 0.99997 and 0.9979 on the PPI network dataset and GraphSAGE-PPI dataset, respectively. Our further results show that the LPPI is promising for large-scale PPI prediction in both accuracy and efficiency, which is beneficial to other large-scale biomedical molecules interactions detection.

## Introduction

Over the past years, with the rapid development of biomedical researches as well as computer technologies, an increasing number of biomedical data, such as biomedical entities and their relationships, have been extracted from unconstructed data (Su et al., 2018). As an important and pervasive data carrier, a graph is considered the most suitable structure to present biomedical entities and their relationships. Both the availability of biomedical data and the researches of graphs have greatly facilitated biomedical graph studies, such as graph embedding, node properties prediction, and link prediction.

As the material basis of life, proteins are involved in every cell and almost every primary cellular process (Gavin et al., 2002). Analyzing protein–protein interactions (PPIs) can provide valuable insights into the molecular mechanisms underlying a living cell (Ma et al., 2011). Due to the rapid research in high-throughput technologies and biomedical studies, millions of PPI data have been collected from various experiments. Many databases have been constructed accordingly. However, too much data brings a few problems, such as high false-positive rates, low coverage, and high cost. Therefore, it is very meaningful to propose a high-efficiency computing method to identify PPIs.

Much work has been done in predicting PPIs. According to the method, it generally can be categorized into two groups based on either (1) feature extraction or (2) based on machine learning and deep learning. For the first group, they concentrate on the feature design. Features are extracted from kinds of sources, including protein sequence, functional domain information, physicochemical properties, and the fusion of feature sources. For example, Shen et al. (2007) predicted PPIs using conjoint-triad feature extracted from protein amino acids to represent protein. His work achieved a promising accuracy of 83.90% when applied to a 16,000 diverse PPI pairs dataset. On the basis of a protein sequence, functional domain information was necessary for the understanding of biological processes. Hence, Mudita and Resat (2007) proposed a method based on quantitative score measuring domain-domain interactions derived from available PPI database, then used the obtained score to predict interaction probability between two proteins. Chen et al. (2019) designed three types of protein-pair features based on physicochemical properties of amino acids, gene ontology annotations, and interaction network topologies. Then they introduced an ensemble learning approach for PPI prediction integrating three kinds of features. As for the second group, they concentrate on the design of classifier or neural network. Both machine learning methods and deep learning methods are based on statistics theories. Machine learning methods utilize classifiers to predict PPIs, such as naïve Bayes (NB), logistic regression (LR), random forest (RF), and support vector machine (SVM). Methods based on deep learning tend to apply neural networks to address PPI prediction, such as convolution neural network (CNN), recurrent neural network (RNN), and long short-term memory (LSTM). For instance, Romero-Molina et al. (2019) predicted the protein-protein interactions using SVM based on the sequence of proteins. Wang et al. (2017a,b,c) explored the protein evolutionary features from the angle of the image processing techniques in order to open a new way of researching protein sequences. Sequence-based approaches typically represent protein sequence as a vector using feature representation method, then the vector as an input of classification algorithm. All of these methods have achieved a promising result. However, they tend to concentrate on protein feature extraction and the design of neural networks and not the complex relationships that the proteins have, such as graph topology. More specifically, proteins collaborate and interact with each other to perform biological functions, leading to many protein interactions, which can be integrated and modeled as a graph/network structure. Therefore, it is important to detecting PPIs from the perspective of graph structure.

Analyzing and modeling the biomedical data with graph structure rely on a thorough understanding of graph topology. Numerous network-based learning methods have been developed to explore the interactions between proteins. They are classified into three categories, based on (1) network diffusion, (2) handcrafted graph features, and (3) graph representation learning. For the first group, the diffusion methods employ random walk techniques for influence propagation in different networks, such as integrating PPI networks into disease gene prediction (Luo et al., 2019). For the second group, various features for proteins are extracted and then fed into traditional machine learning methods. Other tasks also benefit from various features, especially when processing graph structure data. For example, graph clustering task (He et al., 2019a,b) utilize these multiview features to detect biological module. Graph clustering is also conducive to graph representation learning tasks because such methods are able to decrease the graph scale, and they can then improve the efficiency of the representation learning model. As for the third group, instead of a handcrafted feature, graph representation learning methods learn features automatically. This kind of method aims to learn a low-dimension representation for each node. Representative methods include Matrix Factorization-based model, Random Walk-based model, and Neural Network-based model. MF-based model (Belkin and Niyogi, 2003) learns graph representation by factorizing the matrix of input data into lower dimensional matrices. RW-based model (Perozzi et al., 2014; Grover and Leskovec, 2016) learns representation by generating a sequence of nodes randomly. The NN-based model integrates neural networks into representation learning. For example, (Kipf and Welling, 2016) proposed that graph convolutional networks (GCN) are perhaps the most representative graph neural network models, having a strong ability in the task of semi-supervised classification. The key issue in GCN is about the filter design in fact since it has a huge influence on the efficiency of model. Additionally, with the widely used of attention mechanism, attention-based graph neural network born, namely graph attention networks (GATs; Velikovi et al., 2017). Compared with GCN, GATs are more flexible and efficient since less parameters are used and can be parallelized. Although graph embedding is the most popular among these three methods, it usually suffers from high computational and space cost, owing to high dimensionality, sparsity of the network, and rapid expansion of the network. Therefore, developing an efficient framework, which can accelerate graph embedding and improve the embedding results accuracy, is important to both PPI and other molecular interactions.

In this paper, we proposed a multi-level model LPPI to improve both the quality and speed of large-scale PPIs prediction. LPPI consists four parts: (i) data collecting, (ii) graph embedding, (iii) embedding enhancement, and (iv) results prediction. Data collecting contains attribute feature extracting. We adopt the fundamental information as the attribute feature, such as positional gene sets, motif gene sets, and immunological signatures. In addition, the protein attribute is used to reconstruct a weighted graph by calculating node similarity. Then, graph embedding is used to learn the topology feature for each node. During this process, GraphZoom (Deng et al., 2019) is applied to accelerate the embedding process by reducing the size of the graph. After enhancing embedding, the classifier is used to predict interactions between protein pairs.

Our contributions are 2-fold. Firstly, LPPI integrates protein attribute into graph embedding task. More than that, LPPI adds weight to the link by calculating node similarity adopting the protein attribute. In this way, multi-view information is used when learning node representation, which is conducive to the improvement of accuracy. Secondly, we reconstruct the graph by using the GraphZoom algorithm in order to reduce the size of the graph. In this way, we can accelerate the efficiency of any network embedding algorithms. By combining the above two aspects, LPPI can save execution time without losing accuracy. Experiments on PPI network dataset and GraphSAGE-PPI dataset demonstrate that LPPI compares favorably both in classification accuracy and efficiency (measured in CPU time) against baseline models for large-scale PPI prediction.

## Materials and Methods

### Benchmark Dataset

In order to validate the efficiency of our model, we collected two datasets with different sizes, which are the PPI network dataset and the GraphSAGE-PPI dataset. The statistics of the datasets are in Table 1 in which the Density is defined as

$$\frac{2*\#Links}{\#Nodes^{2}}$$

**Table 1:** Statistics of the datasets.

Dataset	#Nodes	#Links	Density
PPI network	23,997	663,954	0.23%
GraphSAGE-PPI	6,370	186,421	0.92%

The positive PPI network dataset was downloaded from Stanford Large Network Dataset Collection (PPI Network, May 2017 version). This version of the PPI Network contains 818,716 protein-protein pairs of experimentally verified PPIs from 23,997 different human proteins. After eliminating self-interactions and duplicate interactions, we finally obtain 663,954 unique positive protein-protein pairs. The dataset is available at http://snap.stanford.edu/graphsage/ppi.zip.

The positive GraphSAGE-PPI (Sep 2018 version) dataset was collected following (Hamilton et al., 2017), which was also constructed by Stanford University. This version data set was used as the benchmark to train GraphSAGE. The data resource was the same as the PPI network. However, differently from PPI network, GraphSAGE-PPI contains fewer nodes and links, which are numbered at 6,370 and 186,421, respectively. The dataset is available at http://github.com/williamleif/GraphSAGE/example_data.

One of the common ways to construct the negative data set is to consider two proteins with different cellular compartments nor interacting. In this study, we adopted the same strategy to construct a negative dataset for two benchmark datasets. We followed this idea and constructed each benchmark dataset according to the following criteria: (1) the number of negative samples was equal to that in the positive dataset; (2) we constructed a complementary graph; (3) we removed the interactions from the same cellular compartments; and (4) we randomly selected noninteracting protein pairs from the complementary graph. After that, a negative dataset had been constructed, which was trained with a positive dataset together. Five-fold cross-validation was adopted when training the model, and, therefore, a negative dataset was constructed at each fold.

### Protein Attribute Extraction

In order to represent protein nodes, we extracted the protein attribute features following (Hamilton et al., 2017). Using positional gene sets, motif gene sets, and immunological signatures as features, collected from the Molecular Signatures Database (Subramanian et al., 2005). Positional gene sets corresponding to each human chromosome and each cytogenetic band that has at least one gene. There are 326 positional gene sets in total. As for the motif gene sets, they represent potential targets of regulation by transcription factors or microRNAs. The sets consist of genes grouped by short sequence motifs they share in their non-protein coding regions. Immunological signatures represent cell states and perturbations within the immune system. The signatures are generated by manual curation of published studies in human and mouse immunology. Finally, the protein attribute feature is obtained.

### Graph Embedding

Graph embedding methods aim to automatically learn a low-dimensional feature representation for each node in the graph (Wang et al., 2016). Traditionally, a low-dimensional feature is considered as the structural information of the graph. Therefore, it can be used in various downstream tasks. Since the concept of graph embedding proposed, graph embedding methods can be categorized into three groups: MF-based, RW-based, and NN-based (Su et al., 2018; Yue et al., 2019).

For the sake of efficiency improvement, we adapted the RW-based method, which was inspired by the word2vec model (Mikolov et al., 2013). The RW-based method tries to learn node representation by generating node sequence through random walk in graphs. In this way, topological information can be preserved into a low-dimensional vector. As the two representative methods based on random walk, DeepWalk (DW; Perozzi et al., 2014) and Node2vec (Grover and Leskovec, 2016) were applied to learn latent features. DW considers the paths as sentences and implements Skip-Gram to learn the embedding of each node. Specifically, the DeepWalk algorithm first generates a random walk path $P_{v_{i}}^{1},P_{v_{i}}^{2},P_{v_{i}}^{3},\ldots ,P_{v_{i}}^{n}\;$ by taking $v_{i}$ as the root node, the symbol n represents the length of random walk path. Therefore, the aim is to predict the next node according previous sequence:

$$\mathrm{Pr}=(P_{v_{i}}^{m}|P_{v_{i}}^{1},P_{v_{i}}^{2},P_{v_{i}}^{3},\ldots ,P_{v_{i}}^{m-1})$$

However, it is difficult to calculate an order sequence in the experiment. In order to solve this problem, Skip-Gram is used to learn the random walk path. This algorithm does not take the sequence order into consideration but sets a sliding window of length n, using target words to predict context. Therefore, the objective function of optimization is as follows:

$$\underset{p_{v_{i}}^{m}}{\min}-\log\mathrm{Pr}\left(\left\{p_{v_{i}}^{m-1},\ldots ,p_{v_{i}}^{m-n},p_{v_{i}}^{m+1},\ldots ,p_{v_{i}}^{m+n}\right\}|p_{v_{i}}^{m}\right)$$

Compared to DeepWalk, Node2vec introduces the probability of controlling the walk direction. Therefore, the objective function of optimization is as follows:

$$\underset{f}{\max}\underset{u\in V}{\sum }\log\mathrm{Pr}\left(N_{s}\left(U\right)|f\left(u\right)\right)\;$$

In this formulation, u represents the current node and $N_{s}\left(U\right)$ represents the nodes selected by strategy s. In Node2vec, it adapts the breadth-first search (BFS) and the depth-first search (DFS) into the generation process of the random walk sequence by introducing return hyperparameter pand ahead hyperparameter q to control the probability of a walk. The probability α(m,θ) from current node *m* to next node θ is defined as follows:

$$\alpha \left(m,\theta \right)=\{\begin{array}{c} \frac{1}{p},if\;d_{m\theta }=0\\ 1,if\;d_{m\theta }=1\\ \frac{1}{q},if\;d_{m\theta }=2 \end{array}$$

Breadth-first search focuses on neighboring nodes and characterizes a relatively local network representation. DFS reflects the homogeneity between nodes at a higher level. Specifically, BFS explores the structural properties of the graph, while DFS explores the similarity in content or similarity between adjacent nodes.

### GraphZoom

Owing to the scalable of PPIs data, it is essential to accelerate the graph embedding process. In this section, GraphZoom (Deng et al., 2019) is applied to improve the accuracy and efficiency of graph embedding. GraphZoom is a multi-level framework for improving both the accuracy and scalability of unsupervised graph embedding algorithms. There are four components in it: (1) graph fusion, (2) spectral graph coarsening, (3) graph embedding, and (4) embedding refinement.

For the first step, original graph topology and attribute information are combined to construct a weighted graph, which has the same number of nodes as the original graph. Graph topology can be represented by the adjacency matrix $A_{topo}\in R^{N\times N}$, and cosine similarity on attribute feature is used to calculate edge weight $A_{feat}$. Then, the fused graph can be represented by a weighted sum:

$$A_{fusion}=A_{topo}+\beta A_{feat}$$

The second step is spectral coarsening, which is the core part of GraphZoom. In order to improve the embedding speed, a fused graph constructed before is coarsened into a much smaller graph by merging nodes with high spectral similarities. Inspired by signal processing, simple smoothing (low-pass graph filtering) function is applied to *k* random vectors to obtain smoothed vectors for *k*-dimensional graph embedding instead of calculating the eigenvectors of the original graph Laplacian. Gauss-Seidel iteration method is used to solve *k* linear equations to obtain initial random *k*-dimensional feature representation. x represents a random vector calculated by Gauss-Seidel, which is expressed with a linear combination of eigenvectors u of the graph Laplacian. Smoothed vector $\tilde{u}$ is obtained by applying the smoothing function. Then the nodes with a higher spectral affinity $a_{p,q}$ are locally clustered, and a graph with fewer nodes (adjacency matrix) is obtained so repeatedly. This method can be achieved in linear time. The whole process can be formulated:

$$x=\overset{N}{\underset{i=1}{\sum }}\alpha_{i}u_{i}\;\overset{smoothing}{\to }\;\tilde{x}=\overset{n}{\underset{i=1}{\sum }}\tilde{\alpha_{i}}u_{i},n\ll N$$

$$a_{p,q}=\frac{{\left|\left(K_{p,:},K_{q,:}\right)\right|}^{2}}{K_{p,:}{}^{2}K_{q,:}{}^{2}}$$

$$\left(K_{p,:},K_{q,:}\right)=\overset{k}{\underset{t=1}{\sum }}\left(x_{p}^{\left(t\right)}\;\cdot \;x_{q}^{\left(t\right)}\right)$$

As for the third step, any unsupervised embedding methods can be applied to embed the coarsest graph. The last step is embedding refinement. Using Laplace smoothing to map the node representation to each node of the original graph, then embedding representation of the original graph node is obtained. The embedding results can be calculated as follows:

$$E_{i}={\left({\tilde{D}}_{i}^{-\frac{1}{2}}{\tilde{A}}_{i}{\tilde{D}}_{i}^{-\frac{1}{2}}\right)}^{k}{\hat{E}}_{i}={\left({\tilde{D}}_{i}^{-\frac{1}{2}}{\tilde{A}}_{i}{\tilde{D}}_{i}^{-\frac{1}{2}}\right)}^{k}H_{i+1}^{i}E_{i+1}$$

$$\tilde{A}=A+\sigma I,\tilde{D}=D+\sigma I$$

where *A* is the adjacency matrix, *D* is the degree matrix, $H_{i+1}^{i}$ is the graph mapping operator between two coarsening levels i and i+1, and σ is a small value to ensure every node has its own self-loop.

## Results

### Evaluation Criteria

To verifies the proposed method in the experiments, we followed the 5-fold cross-validation specification. To evaluate the proposed method more fairly, a range of performance evaluation measures were computed including accuracy (Acc.), sensitivity (Sen.), precision (Pre.), and the Matthews correlation coefficient (MCC), which can be defined respectively:

$$\mathrm{Accuracy}=\frac{TN+TP}{TN+TP+FN+FP}$$

$$\mathrm{Sensitivity}=\frac{TP}{TP+FN}$$

$$\mathrm{Precision}=\frac{TP}{TP+FP}$$

$$\mathrm{MCC}=\frac{TP\times TN-FP\times FN}{\sqrt{\left(TP+FP\right)\left(TP+FN\right)\left(TN+FP\right)\left(TN+FN\right)}}$$

where the TN, TP, FN, and FP denotes the number of correctly predicted positive and negative samples, wrongly predicted positive and negative samples, respectively. Furthermore, the Receiver Operating Characteristic (ROC) curve, which represents the results of multiple confusion matrices, using a false positive rate as its *x*-axis and true positive rate as its *y*-axis. The area under curve (AUC) of ROC, which follows a philosophy of the bigger the better, is also adopted to measure the performance of the proposed model.

### Model Construction

**Figure 1:**
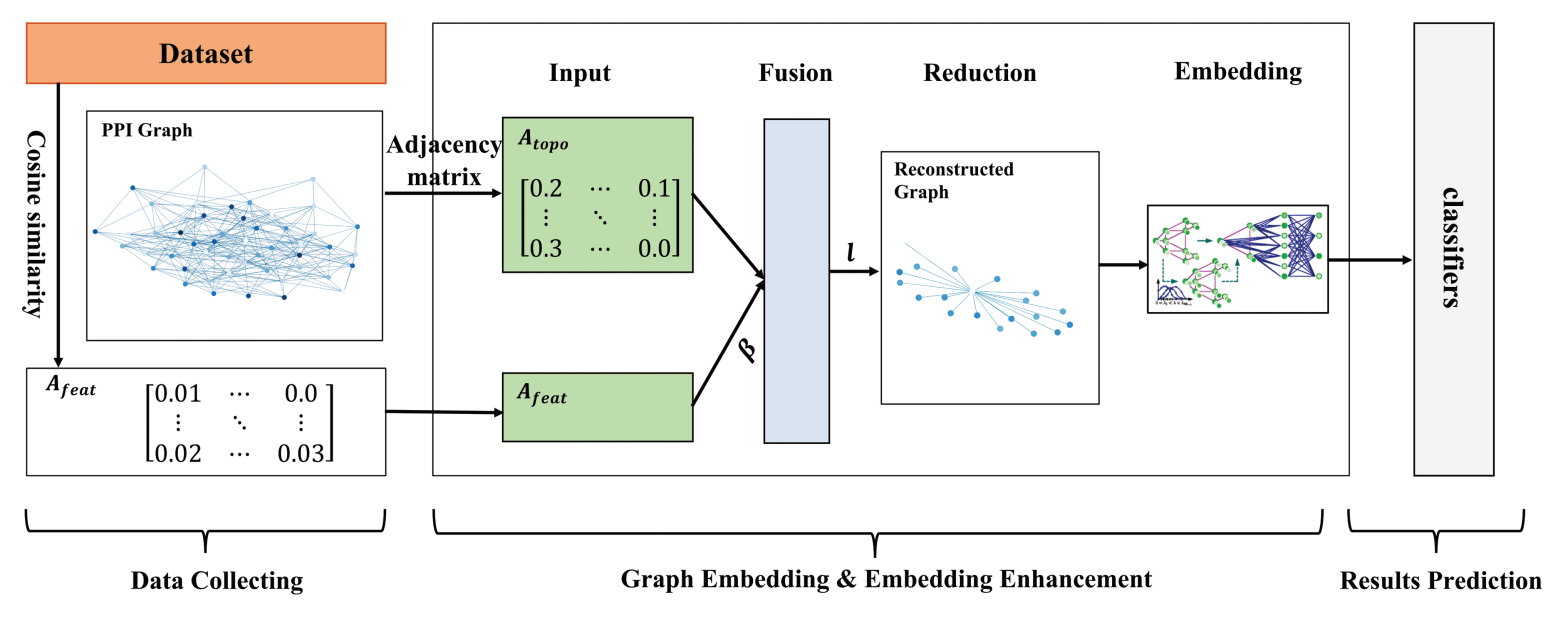
The overview of the proposed model.

We implemented our model on two data sets of different sizes. In order to maintain unity, we also integrated DeepWalk and Node2vec as the basic embedding methods into the proposed model, respectively. As for the hyperparameters in DeepWalk and Node2vec, we used 10 walks with a walk length of 80 and set the embedding dimension to 128. In addition, Node2vec has two parameters, return parameter *p* and in-out parameter *q*, which control the direction of the next step. We set them to 1.0 and 0.5, respectively. The effectiveness of these parameters is verified by other experiments. GraphZoom is used to enhance the graph and accelerate the graph embedding process. The hyperparameters used in GraphZoom are fusion parameter β and coarsening level l. β controls the proportion of attribute feature. Parameter l is used in the graph reduction process, which controls the size of the reconstructed graph. Coarsening level *l* represents the iteration that the original graph is to be reconstructed. With the increase of the coarsening level, the scale of the graph is smaller. We adapted 0.1 and 1 in the baseline model, respectively. After obtaining graph embedding representation, several classifiers were applied to predict protein pairs. It should note that all parameters used in classifiers were the default. The model overview is shown in Figure 1.

### Performance on Two Large-Scale Datasets

**Figure 2:**
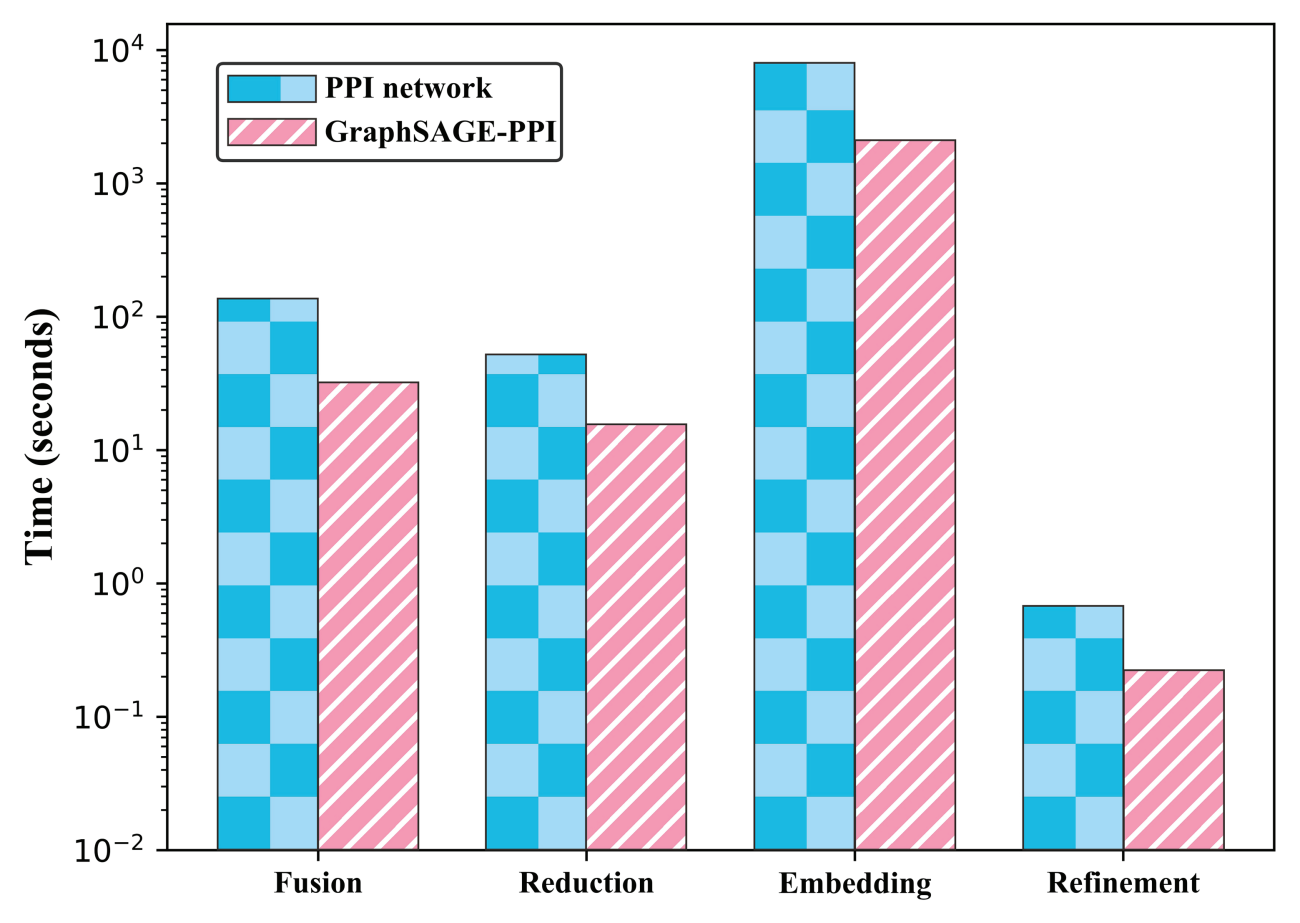
Timing experiments of four parts on PPI network dataset and GraphSAGE-PPI dataset.

We test the performance of our model on two benchmark datasets. To contextualize the empirical results on benchmarks, we construct a baseline model, which integrates DeepWalk (Perozzi et al., 2014) as the graph embedding method and LR (Hosmer et al., 2013) as a classifier. Five-fold cross-validation is used to test the baseline model. The results are shown in Table 2. In addition, we also compare the CPU time of two datasets, which is shown in Figure 2.

**Table 2:** Prediction results for two datasets. DW means Deepwalk, and LR represents Logistic Regression.

Baseline model	Fold	PPI network					GraphSAGE-PPI				
		Acc.	Pre.	Sen.	MCC	AUC	Acc.	Pre.	Sen.	MCC	AUC
LPPI (GZ-DW-LR)	0	0.99996	1.0	0.99992	0.99992	0.99996	0.9978	1.0	0.9956	0.9957	0.9978
	1	0.99997	1.0	0.99993	0.99993	0.99997	0.9979	1.0	0.9958	0.9958	0.9979
	2	0.99995	1.0	0.99991	0.99991	0.99996	0.9981	1.0	0.9961	0.9961	0.9981
	3	0.99997	1.0	0.99993	0.99993	0.99997	0.9980	1.0	0.9960	0.9959	0.9980
	4	0.99998	1.0	0.99996	0.99996	0.99998	0.9978	1.0	0.9957	0.9956	0.9978
Average		**0.99997**	**1.0**	**0.99993**	**0.99993**	**0.99996**	**0.9979**	**1.0**	**0.9958**	**0.9958**	**0.9979**

The bold values mean the best results achieved.

Our model achieves a highly predictive performance on both two datasets, which average accuracy is 0.99997 and 0.9979, respectively. Compared with GraphSAGE-PPI data et, our model achieves better results on the PPI network dataset, which demonstrates that our model has the ability to process large-scale dataset precisely. More specifically, the number of nodes and links of the PPI network dataset is three times that in the GraphSAGE-PPI dataset in fact; however, the time cost of two datasets is similar, which further demonstrates that our model can process the large-scale dataset efficiently. In conclusion, the proposed model has the ability to process high-density network both accurately and efficiently.

### Comparing LPPI With Baseline Embedding Methods

**Figure 3:**
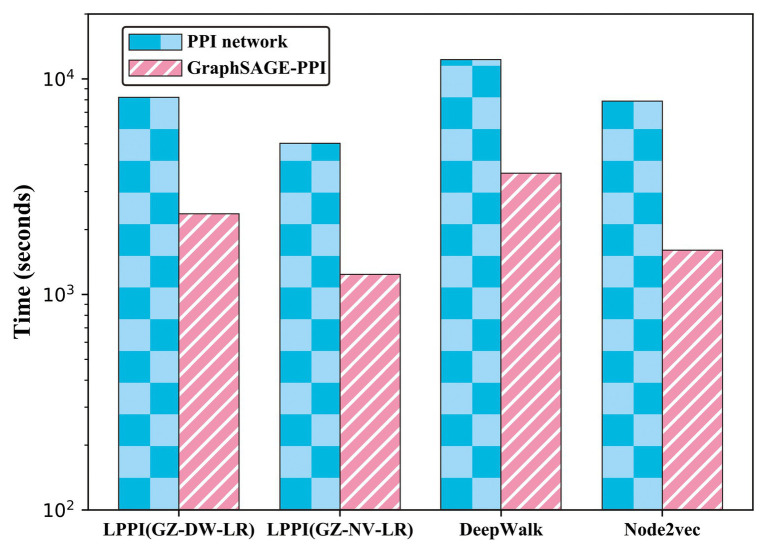
Timing experiments of different embedding methods on PPI network dataset and GraphSAGE-PPI dataset.

In order to validate that the proposed model accelerates the embedding process without losing accuracy, we compared the proposed model with two baseline embedding methods, which are also integrated into the proposed model as part of the embedding. The results are shown in Table 3 and Figure 3.

**Table 3:** Summary of results in terms of mean classification accuracy (Acc.), AUC, and CPU time for different combinations in LPPI on the PPI network dataset and GraphSAGE-PPI dataset.

Method	PPI network			GraphSAGE-PPI		
	Acc.	AUC	Time(s)	Acc.	AUC	Time(s)
LPPI (GZ-DW-LR)	**0.99997**	0.99996	8131.417	0.9979	0.9979	2309.847
LPPI (GZ-NV-LR)	0.99993	**0.99997**	**5001.137**	**0.9984**	0.9983	**1232.644**
DeepWalk	0.99975	0.99990	12405.259	0.9544	0.9995	3633.228
Node2vec	0.99992	0.99995	7947.544	0.9879	**0.9999**	1580.749

The bold values mean the best results achieved.

According to the results, firstly, the proposed model used less CPU time since LPPI had the graph reduction module, which can decrease the graph scale. In addition, it can be observed that the proposed model achieved higher accuracy than the other two baseline models on both the PPI network dataset and the GraphSAGE-PPI dataset. This is because LPPI contains more detailed information such as node attribute and concentrates more on the key part of the graph and eliminates noisy information. In conclusion, the proposed model performs better than baseline models mainly because (i) LPPI integrates node attribute information and node similarity as topology information into the model, which increase the accuracy of the proposed model, and (ii) LPPI reconstructs graph to reduce the graph scale, which is conducive to efficiency on embedding and noisy information eliminated.

### Analysis on LPPI Kernels

**Figure 4:**
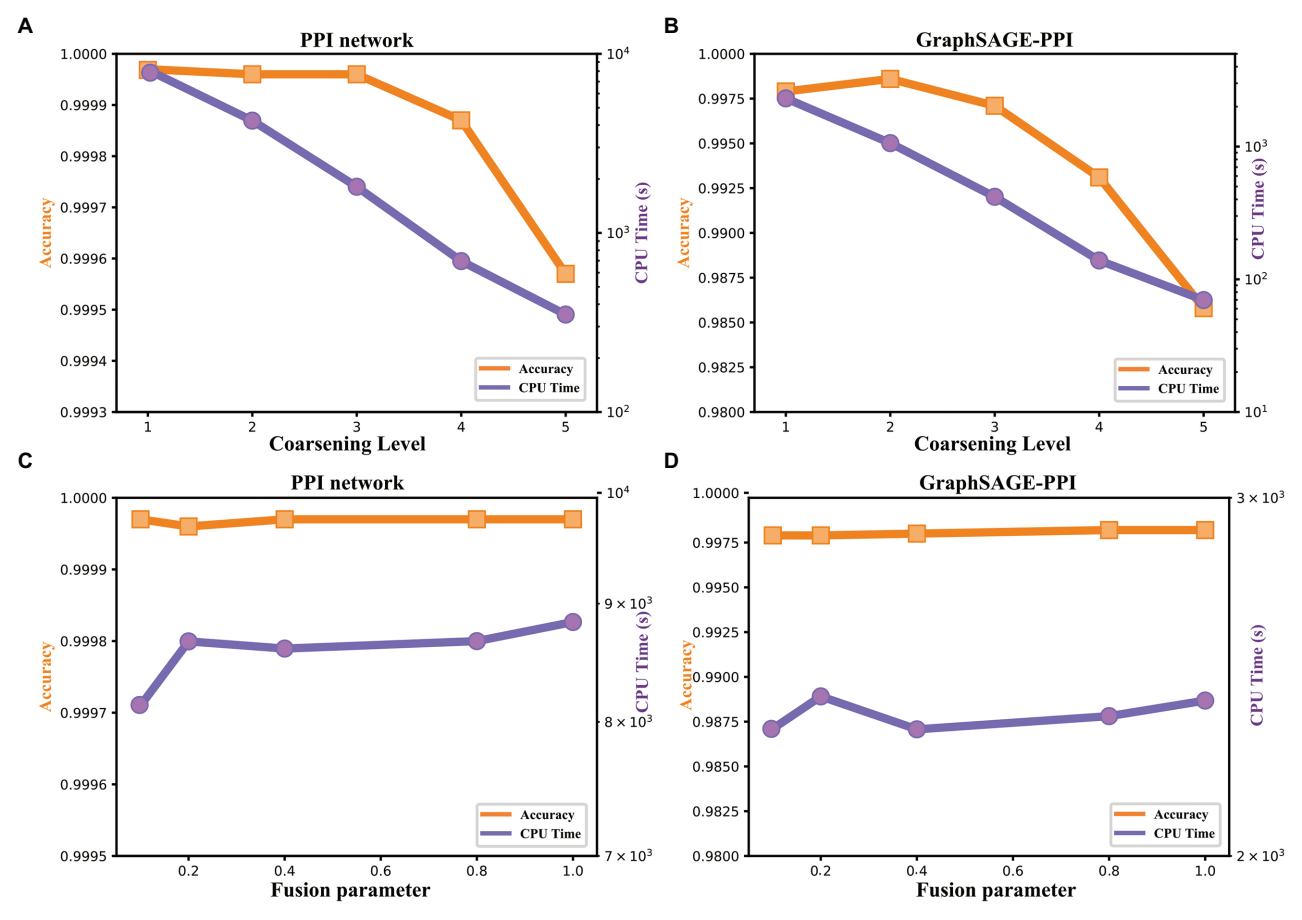
Accuracy and timing experiments on two benchmark datasets. **(A)** Model performance with respect to the coarsening level on PPI network dataset. **(B)** Model performance with respect to the coarsening level on the GraphSAGE-PPI dataset. **(C)** Model performance about fusion parameter on PPI network dataset. **(D)** Model performance about fusion parameter on the GraphSAGE-PPI dataset.

There are two hyperparameters in LPPI model, which are fusion parameter β and coarsening level parameter l. In order to study the efficiency and accuracy of LPPI, we focused on two parameters. The results are shown in Table 4 and Figure 4.

**Table 4:** Comparisons of different kernel parameters in GraphZoom in classification on the PPI network dataset and GraphSAGE-PPI dataset.

Method	PPI network			GraphSAGE-PPI		
	Acc.	AUC	Time(s)	Acc.	AUC	Time(s)
DeepWalk	0.99975	0.99990	12405.259	0.9544	0.9995	3633.228
LPPI(DW-LR,*l* = 1)	**0.99997**	**0.99996**	8131.417 (×1.5)	0.9979	0.9979	2309.847 (×1.6)
LPPI(DW-LR,*l* = 2)	0.99996	0.99996	4236.696 (×2.8)	**0.9986**	**0.9985**	1062.251 (×3.4)
LPPI(DW-LR,*l* = 3)	0.99996	0.99996	1810.727 (×6.9)	0.9971	0.9971	418.485 (×8.7)
LPPI(DW-LR,*l* = 4)	0.99987	0.99985	696.115 (×17.8)	0.9931	0.9931	138.625 (×26.2)
LPPI(DW-LR,*l* = 5)	0.99957	0.99957	**350.093 (×35.4)**	0.9858	0.9856	**69.745 (×52.1)**
LPPI(DW-LR,*β* = 0.1)	**0.99997**	**0.99996**	**8131.417 (×1.5)**	0.9979	0.9979	**2309.847 (×1.6)**
LPPI(DW-LR,*β* = 0.2)	0.99996	0.99980	8667.839 (×1.4)	0.9979	0.9979	2396.033 (×1.5)
LPPI(DW-LR,*β* = 0.4)	**0.99997**	**0.99996**	8606.011 (×1.4)	0.9980	0.9978	2318.294 (×1.6)
LPPI(DW-LR,*β* = 0.8)	0.99997	0.99997	8669.954 (×1.4)	**0.9982**	0.9982	2342.992 (×1.6)
LPPI(DW-LR,*β* = 1.0)	0.99997	0.99995	8836.558 (×1.4)	**0.9982**	0.9981	2384.745 (×1.5)

The bold values mean the best results achieved.

**Figure 5:**
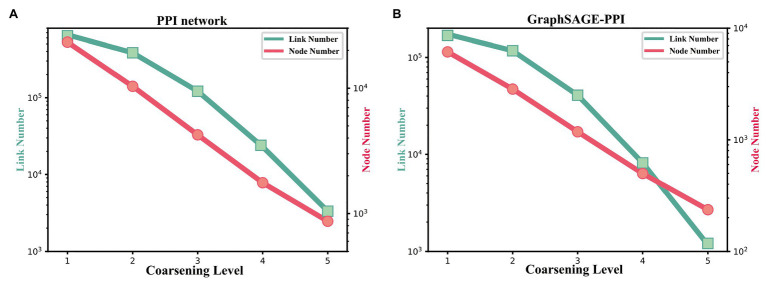
(A) The change of link number and node number with the coarsening level increasing on the PPI network dataset. (B) The change of link number and node number with the coarsening level increasing on the GraphSAGE-PPI dataset.

Firstly, we discuss the influence of coarsening level. Coarsening level controls the size of the reconstructed graph. Figure 5 shows that the bigger the coarsening level is, the smaller the reconstructed graph is. In our experiment, five different values are used. From the results, we can know that with the increase of the coarsening level, the accuracy of the two datasets is gradually decreased from 0.99997 to 0.99957 and from 0.9979 to 0.9858, respectively. Correspondingly, the CPU time is dramatically decreased from 8131.417 to 350.093 s and from 2309.847 to 69.745 s, respectively. When the coarsening level is 1, the model achieves the highest accuracy on the PPI network dataset, which is 0.99997, but it costs the most CPU time. The model with coarsening level 5 is the most efficient model as it costs the least CPU time, which is 350.093 s. More importantly, though the model using level 5 has the lowest accuracy, its accuracy is not much different from the model with level 1. As for the GraphSAGE-PPI dataset, LPPI achieves the best performance when the level is 2 with an accuracy of 0.9986 and AUC value of 0.9985. Overall, when the number of coarsening level is less than 5, the accuracy of LPPI is always higher than that of DeepWalk and LPPI improves the efficiency of DeepWalk by 17.8 times and 26.2 times on two datasets, respectively. Hence, experiment results prove that our model can accelerate the embedding process without losing accuracy.

Next, we discuss the fusion parameter β, which decides the proportion of attribute feature. In this part, we also try five different values for parameter β. According to our experiment results (Figures 4C,D), this parameter has a positive influence on the final result. With the increase of β, the accuracy is increase gradually. For the PPI network, the highest accuracy is 0.99997, which is achieved by 0.1, 0.8, and 1. As for the GraphSAGE-PPI, the highest accuracy is obtained when β is 0.8 and 1. This result indicates that combing the attribute feature with network embedding can improve the predictive performance. In addition, CPU time has not been affected by parameter β as this parameter has no influence on the scale of the reconstructed graph. Even though, CPU time cost by LPPI with various fusion parameter is still less than that of DeepWalk.

In this part, we discuss two parameters used in our model. Parameter coarsening level l can accelerate the embedding process, parameter β can improve the accuracy value. These two parameters further demonstrate that our model has the ability to balance the performance of accuracy and efficiency.

### Comparison of Different Classification Algorithms

After obtaining embedding features, classifiers are used to classify the protein pairs. In this section, we compare the results of different classifiers. Base on the baseline model, we compare three types of classifiers, including LR, RF, and NB (Rish, 2001; Liaw and Wiener, 2002; Hosmer et al., 2013) and the predictive performance is shown in Table 5. It should note that default parameters are used in different classifiers.

**Table 5:** Comparisons of different classifiers on the PPI network dataset and GraphSAGE-PPI dataset.

Method	PPI network			GraphSAGE-PPI		
	Acc.	AUC	Time (s)	Acc.	AUC	Time(s)
LPPI(GZ-DW-LR)	0.99997	0.99996	**8131.417**	0.9979	0.9979	**2309.847**
LPPI(GZ-DW-RF)	**0.99999**	**0.99998**	17783.854	**0.9999**	**0.9999**	2874.321
LPPI(GZ-DW-NB)	0.98799	0.99996	17673.404	0.9899	0.9956	2821.121

The bold values mean the best results achieved.

In our experiment, we test classifiers based on LPPI (GZ-DW). Among these three classifiers, LR is a linear model, RF belongs to an ensemble-based model, and NB is a generation model. From the results, it can be found that though RF achieves the best performances on both accuracy and AUC value for each dataset, it costs the longest time, which is not suitable for the sake of efficiency. On the other hand, LR has not only a promising performance with high accuracy and the AUC, but the least CPU time. As a result, LR is selected as the final classifier integrated into LPPI.

## Discussion

The proposed model has promising predictive performances on two large-scale datasets, the PPI network dataset and GraphSAGE-PPI dataset, which have 663,954 links and 186,421 links in total, respectively. Our model aims to address large-scale protein pairs prediction, efficiently and accurately. However, it is introductive to point out that there are still several limitations in our model. The current study constructs a multi-level framework for PPI prediction, containing four parts. In fact, classifiers as well as parameters affect results significantly, especially in classification tasks. Therefore, the performance of our model could still have a bias. Simultaneously, a multi-level framework is not convenient for a training model. In order to solve this problem, an end-to-end model is expected to be adapted. More specifically, we can replace classify layer with a forward neural network, which contributes to model training and CPU time. In addition, from the perspective of code implement, it is not efficient enough to link prediction tasks since the code is not parallelized, such as in the part of split data and 5-fold cross-validation.

Future efforts to improve the prediction of PPI based on the current study include (i) reducing the bias caused by classifiers, replacing the classify layer with a forward neural network, and (ii) improving efficiency through parallel computing, especially in the part of graph embedding.

## Conclusion

In this study, we introduce a model LPPI, a multi-level framework to improve the accuracy and efficiency of large-scale protein-protein interactions prediction. The attribute feature is collected in LPPI firstly, which further is used to calculate the similarity between protein nodes to reconstruct a weighted graph. Then, a graph embedding method, such as DeepWalk and Node2vec, is applied to a new graph and generates topology features. Afterward, the classifier is used to test if protein pairs interact with each other. Experiments show that LPPI improves both classification accuracy and embedding speed on two benchmark datasets. Our work provides a new framework for large-scale protein-protein interactions prediction, which is beneficial to the detection of other biomedical molecule interactions.

## Data Availability Statement

Publicly available datasets were analyzed in this study. This data can be found at: https://github.com/Blair1213/LPPI.

## Author Contributions

X-RS and Z-HY designed the model and wrote the manuscript. X-RS, LH, Y-AH, YW, and H-CY conducted the experiments. Z-HY managed and directed the project. All authors contributed to the article and approved the submitted version.

### Conflict of Interest

The authors declare that the research was conducted in the absence of any commercial or financial relationships that could be construed as a potential conflict of interest.
